# Think Before You Ink: Perception, Prevalence, and Correlates of Tattooing and Tattoo Regret in US Adults

**DOI:** 10.7759/cureus.48167

**Published:** 2023-11-02

**Authors:** Robert Morlock, Amy Morlock

**Affiliations:** 1 Health Services Research, Acumen Health Research Institute, Ann Arbor, USA

**Keywords:** quality-of-life, perception, regret, correlates, prevalence, body art, tattoo

## Abstract

Introduction

Few population-based studies have examined the perception and prevalence of tattoos and tattoo regret in the general United States (US) adult population. Our objective was to report the perception of people with tattoos and describe the prevalence, socio-demographics, health-related quality of life, and the extent of tattoo regret in US adults.

Methods

Data were assessed from a cross-sectional study of US adults. Participants were recruited using a random stratified sampling framework similar to the US Census. Data collected for all participants included socio-demographic and clinical characteristics, general health-related quality of life (Veterans RAND 12‐item), depression (Patient Health Questionnaire 9-item), anxiety (Generalized Anxiety Disorder 7-item), and perceptions of those with tattoos. Those with tattoos also answered questions about their tattoo(s), including age when first tattooed, reasons for getting a tattoo, and tattoo regret. Categorical data were described by percentages, and continuous data by mean and standard deviation. Proportions were compared with Chi-squared tests and the means with ANOVA. A logistic regression controlling for confounding variables was carried out to assess factors predictive of tattoo regret.

Results

Of the 3033 participants, 35.3% (1,072) reported having a tattoo. Those more likely to have a tattoo were female (58% vs. 45%), younger (38 vs. 46 years), smoked cigarettes (38% vs. 19% non-smoker), and/or reported an alcohol or drug problem (10% vs. 5%). Those without tattoos were more likely to perceive those with tattoos as less attractive, intelligent, professional, and more rebellious. More time (in years) with a tattoo, having a tattoo on the face, neck, hands, wrist, or fingers, getting a tattoo because of peer pressure, being impaired when getting a tattoo, and experiencing an adverse event related to a tattoo were predictive of tattoo regret. Older age and remembrance as the reason for a tattoo were predictive of not having tattoo regret.

Conclusion

More than one-third of the study sample comprised of adults in the US reported having at least one tattoo. While most people, regardless of their tattoo status, perceived tattooed and non-tattooed individuals equally, tattooed individuals were more likely to be perceived negatively than positively by those without tattoos. Whether tattooed or not, being aware of varying perceptions of tattoo status may be helpful in facilitating positive outcomes.

## Introduction

Since the early 2000s, the prevalence of tattoos in industrialized countries has been reported to be approximately 10% to 20% and rising [1]. In 2019, Ipsos published data indicating an increase in tattoo prevalence in US adults from 21% in 2012 to 30% in 2019 [2]. Ipsos further notes that prevalence is much higher in younger adults: 40% of those aged 18-34 years versus 16% of those aged 55 years and older [2]. Previous studies have noted differences in tattoo prevalence by age [3,4]. Motivation for obtaining a tattoo varies, although often it represents a means of self-expression or symbolic meaning [5,6]. Some may seek tattoos to improve self-esteem; some recipients report lower levels of appearance dissatisfaction immediately following receipt of a tattoo [7].

Among those who obtain tattoos, some often experience regret and/or seek to remove or alter one or more tattoos [6,8-10]. Changes in the attributed meaning of tattoos, the incidence of tattoo-related medical complications, and social or career-related feedback can lead to tattoo regret [6,8,10]. Historically, tattoos have held negative connotations, including promiscuity, mental illness, substance abuse, and decreased intelligence [11], although more recent research suggests that negative perceptions, particularly with regard to employment, are either no longer relevant or are dependent on multiple factors (e.g., type and location of tattoo, type of job, age of interviewer) [12,13]. While considered safe by many, tattooing is also considered an invasive procedure that can result in complications and adverse events (AEs) [14,15].

While some studies find no significant differences in regret by demographic or tattoo characteristics [10], others report that tattoos obtained during teenage years are the most often regretted [16]. Some suggest this may be due to adolescents making more immature, emotionally-driven decisions regarding tattoos [17]. Previous studies of tattoo regret often focus on specific geographic areas [9,10,17] or within specific sub-populations, like adolescents [17] or military veterans [5]. To add to the body of research on tattoos and tattoo regret, we sought to report the perception of people with tattoos and describe the prevalence, socio-demographics, health-related quality of life, and the extent of tattoo regret in a representative sample of US adults.

## Materials and methods

This study is based on data collected in 2017 through a cross-sectional online survey of the general US adult population. A random stratified sampling framework ensured the sample's demographic composition was similar to the US adult population by sex, age, race, and region, according to the US Census Bureau 2011-2015 American Community Survey five-year estimate. The inclusion and exclusion criteria included residing in the US, being over 18 years of age and consenting to participate in the survey. Survey participants were recruited from Acumen Health Research Institute's (AHRI's) research panel and compensated for their time. Before participating in the study, participants read an online consent form explaining the study, and only participants agreeing to participate went on to complete the survey. Established quality control measures, which included paying attention questions, flatline respondent assessments, and consistency questions, were used to identify and remove fraudulent responses. All data were self-reported. As linkable personally identifying data was not collected from participants, the project was found to be exempt from Institutional Review Board approval.

The study questionnaire asked for information on socio-demographics, comorbid conditions, HRQoL, risk-taking behaviors, and tattoo-related questions. Validated instruments included the Veterans RAND 12-item Health Survey (VR-12) [18], the Patient Health Questionnaire (PHQ-9) [19], the Generalized Anxiety Disorder 7 (GAD-7) [20], and the Alcohol Use Disorders Identification Test (AUDIT-C) [21]. The survey was available only in American English.

Socio-demographics included age, sex, race, ethnicity, marital status, education, body mass index (BMI), and comorbid conditions. Risk-taking was assessed using the AUDIT-C, a question asking about the use of recreational drugs (yes or no), and a question asking about time spent in jail (never, less than three days, or more than three days). Alcohol consumption was classified as no hazardous drinking, hazardous drinking (an AUDIT-C score of three for adult women or a score of four or more for adult men), and alcohol use disorder (AUD) (an AUDIT-C score of four or more for adult women or a score of five or more for adult men) [21].

The PHQ-9 is used to screen, diagnose, assess, and monitor for depression. The PHQ-9 also provides an assessment of symptom severity. Scores range from 0 to 27: 0 no depression; 1 to 4 minimal depression; 5 to 9 mild depression; 10 to 14 moderate depression; 15 to 19 moderately severe depression; and 20 to 27 severe depression [19].

The GAD-7 screens for and assesses the severity of anxiety [20]. Scores range from 0 to 21: 0 No anxiety; 1 to 4 minimal anxiety; 5 to 9 mild anxiety; 10 to 14 moderate anxiety; and 15 to 21 severe anxiety.

HRQoL was assessed using the VR-12, which measures a mental and physical component score (MCS and PCS) [18]. PCS and MCS summary scores are standardized using a t-score transformation and normed to a US population with a score of 50 and a standard deviation of 10. Scores range to 100, with higher scores indicating better physical or mental health. The VR-6D is an overall health utility metric derived from the VR-12, where scores generally range from 0 for the worst health state or death to 1 for the best health state [22].

All participants (those with and without tattoos) were asked to assess the perceived attributes (i.e., rebelliousness, attractiveness, intelligence, and professionalism) of those with vs. without tattoos on a scale of one to three (1=less; 2=equally; 3=more). Tattooing status was assessed using the question: "Do you have or have you had a tattoo?" with the response options "no" or "yes." Participants with tattoos were asked several questions about their tattoo(s) including the number of tattoos, size (i.e., larger than their palm) and location of the tattoo(s), reason for receiving tattoo(s), if they were impaired (i.e., drunk or high) when receiving tattoo(s), if they regret their tattoo(s), and the age they received their first tattoo. Additionally, those with tattoos were asked if they experienced an AE such as infection, pain, edema, or pruritus around the tattoo site. Finally, participants were asked about their future plans for tattoos (e.g., removing, modifying, getting more, or no interest in changing tattoo status).

Statistical analyses

Univariate descriptive statistics and frequency distributions were undertaken for every variable. Bivariate comparisons on continuous variables and scales were conducted using t-tests for comparisons between two groups and ANOVA for comparisons with three groups or more. Chi-squared tests were conducted in the case of categorical variables.

A multivariate logistic model assessed tattoo regret (i.e., regretting some or all tattoos) vs. no tattoo regret. Covariates in the model included age, marital status, sex, number of comorbid conditions, education, employment, time with tattoo, an indicator for impairment when getting a tattoo, location of tattoo, reason for getting the tattoo, and an indicator for experiencing a tattoo-related AE. All analyses were performed using SPSS version 28 (IBM Inc., Armonk, New York).

## Results

Comparing the study population to the general US population

The survey had a response rate of 82.6%, with 3,033 participants responding to 3,672 survey invitations. The sample population was approximately similar to the 2011-2015 US Census American Community Survey five-year estimates of population for region, sex, race, and age. A comparison of the study population to the US Census population is provided in Table 1.

**Table 1:** Characteristics of the study population vs. the US population

Characteristics	Study Population	2011-2015 US Census
	N=3033 (100.0%)	N=237,794,858 (100.0%)
Region, N (%)		
Northwest	609 (20.1)	43,135,987 (18.1)
Midwest	672 (22.2)	50,953,438 (21.4)
South	1110 (36.6)	89,030,395 (37.4)
West	642 (21.2)	54,669,038 (23.0)
Sex, N (%)		
Female	1505 (49.6)	122,178,998 (51.4)
Male	1528 (50.4)	115,615,860 (48.6)
Race, N (%)		
Black or African-American	381 (12.6)	29,439,003 (12.4)
White	2385 (78.6)	182,959,364 (76.9)
All other races or more than one race	267 (8.8)	25,396,491 (10.7)
Of Hispanic, Latino, or Spanish origin, N (%)		
Yes	259 (8.5)	36,477,731 (15.3)
No	2774 (91.5)	201,317,127 (84.7)
Age categories, N (%)		
18-44 years	1678 (55.3)	111,597,127 (46.9)
45-64 years	976 (32.2)	81,991,667 (34.5)
65 or more years	379 (12.5)	44,158,505 (18.6)

Comparing those with vs. those without tattoos

Demographic Characteristics

Of the 3,033 participants, 35.3% (n=1072) reported having one or more tattoos. More females than males reported having tattoos (58.1% vs. 41.9%, p<0.001, Table 2). Those with tattoos had a mean age of 8.1 years below those without tattoos (37.9 (SD 12.4) vs. 46.0 (SD 17.0) years; p<0.001), a higher body mass index (BMI) (28.5 (SD 7.0) vs. 27.9 (SD 6.6); p=0.040) and were less likely to have at least a high school education (66.3% vs. 75.5%; p<0.001) compared to those without tattoos. Higher rates of cigarette smoking (38.0% vs. 18.5%; p<0.001) or a self-reported alcohol or drug problem (10.2% vs. 4.7%, p<0.001) were observed for those with tattoos compared with those without.

**Table 2:** Demographic characteristics of those with vs. those without tattoos

Characteristics	Study Population (N=3,033)		p-value
	Without tattoos, N=1961 (64.7%)	With tattoos N=1072 (35.3%)	
Sex, N (%)			
Female	882 (45.0)	623 (58.1)	<0.001
Male	1079 (55.0)	449 (41.9)	
Race, N (%)			
Black or African-American	256 (13.1)	125 (11.7)	0.135
White	1521 (77.6)	864 (80.6)	
All other races or more than one race	184 (9.4)	83 (7.7)	
Age (years), mean (SD)	46.0 (17.0)	37.9 (12.4)	<0.001
Age categories, N (%)			
18-44 years	912 (46.5)	766 (71.5)	<0.001
45-64 years	701 (35.7)	275 (25.7)	
65 or more years	348 (17.7)	31 (2.9)	
Marital status, N (%)			
Divorced or separated	215 (11.0)	147 (13.7)	<0.001
Married or living with partner	1020 (52.0)	587 (54.8)	
Single, never married	661 (33.7)	323 (30.1)	
Widowed	65 (3.3)	15 (1.4)	
Education, N (%)			
Less than a high school diploma	481 (24.5)	361 (33.7)	<0.001
High school diploma or more	1480 (75.5)	711 (66.3)	
Median household income, mean (SD)	$59,018 ($23,567)	$53,608 ($19,072)	<0.001
Weight			
BMI, mean (SD)	27.9 (6.6)	28.5 (7.0)	0.04
Obese, N (%)	616 (31.4)	365 (34.0)	0.138
Exercise, N (%)			
More than 3 times per week	1397 (71.2)	746 (69.6)	0.3
Substance use, N (%)			
Cigarette use	363 (18.5)	407 (38.0)	<0.001
Alcohol or drug problem	92 (4.7)	109 (10.2)	<0.001

Clinical Characteristics

Individuals with tattoos also reported a higher number of medical conditions than those without tattoos (5.1 (SD 3.9) vs. 4.6 (SD 3.8); p<0.05, Table 3), and had significantly higher rates of several conditions, including bipolar disorder (10.0% vs. 4.3%; p<0.001), hepatitis C (2.0% vs. 0.7%; p<0.05), human papillomavirus (HPV) (3.0% vs. 1.4%; p<0.05), insomnia (28.8% vs. 18.2%; p<0.001), migraines or severe headaches (24.5 vs. 16.9; p<0.001), and chronic pain (13.2% vs. 9.2%; p<0.05). Medical conditions that were significantly lower for those with tattoos included hyperlipidemia (14.6% vs. 24.2%; p<0.001) and hypertension (24.0% vs. 32.1%; p<0.001).

Significantly more individuals with tattoos had scores of moderate to severe depression (≥10) on the PHQ-9 than individuals without tattoos (35.3% vs. 22.9%; p<0.001). Similarly, significantly more individuals with tattoos had scores of moderate to severe anxiety (≥10) on the GAD-7 than individuals without tattoos (32.0% vs. 20.2%; p<0.001).

Health-Related Quality of Life

Those with tattoos scored significantly lower (worse) on the MCS of the VR12 (43.1 (SD 12.6) vs. 47.4 (SD 11.9); p<0.001, Table 3). There was no statistically significant difference in physical health as measured by the PCS of the VR12 in those with and without tattoos (46.5 (SD 9.7) vs. 47.3 (SD 9.5); p=0.052). Overall health status as measured with the VR6D health utility measure of the VR12 was lower for those with tattoos vs those without tattoos (0.68 (SD 0.12) vs. 0.72 (SD 0.12); p<0.001).

**Table 3:** Health characteristics of those with vs. those without tattoos GAD-7 - Generalized Anxiety Disorder 7-item; HPV - human papillomavirus; MCS - Mental Health Composite Score; PCS - Physical Composite Score; PHQ-9 - Patient Health Questionnaire; VR6D - Health Utility Score.

Charateristics	Without tattoos	With tattoos	p-value
	N=1961 (64.7%)	N=1072 (35.3%)	
Total medical conditions mean (SD)	4.6 (3.8)	5.1 (3.9)	<0.05
Medical condition			
Bipolar disorder	85 (4.3)	107 (10.0)	<0.001
Hyperlipidemia	475 (24.2)	156 (14.6)	<0.001
Hypertension	630 (32.1)	257 (24.0)	<0.001
Hepatitis C	14 (0.7)	21 (2.0)	<0.05
HPV	28 (1.4)	32 (3.0)	<0.05
Insomnia	356 (18.2)	309 (28.8)	<0.001
Migraines or severe headaches	332 (16.9)	263 (24.5)	<0.001
Chronic pain	180 (9.2)	142 (13.2)	0.001
Depression (PHQ-9), N (%)			
No to mild (PHQ-9≤9)	1511 (77.1)	694 (64.7)	<0.001
Moderate to severe (PHQ-9>10)	450 (22.9)	378 (35.3)	
Depression (PHQ-9), mean (SD)	5.64 (6.58)	7.94 (6.72)	<0.001
Anxiety (GAD-7), N (%)			
No to mild (GAD-7≤9)	1565 (79.8)	729 (68.0)	<0.001
Moderate to severe (GAD-7>10)	396 (20.2)	343 (32.0)	
Anxiety (GAD-7), mean (SD)	5.08 (5.65)	7.20 (5.89)	<0.001
MCS, mean (SD)	47.4 (11.9)	43.1 (12.6)	<0.001
PCS, mean (SD)	47.3 (9.5)	46.5 (9.7)	0.052
Health utility (VR-6D), mean (SD)	0.72 (0.12)	0.68 (0.12)	<0.001

Perceptions of Tattooed Individuals

**Figure 1:**
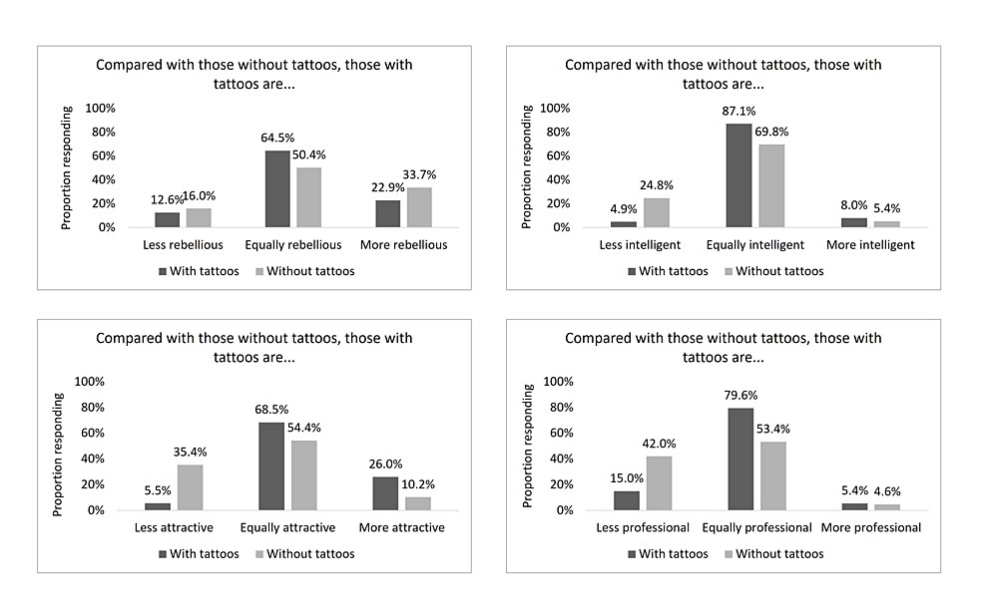
Perceptions of the (A) rebelliousness, (B) attractiveness, (C) intelligence, and (D) professionalism of individuals with tattoos by those with and without tattoos

All participants were asked about their perceptions of those with and without tattoos, including perceptions about rebelliousness, attractiveness, intelligence, and professionalism. Among those without tattoos, 33.7% perceived individuals with tattoos as more rebellious than those without tattoos; among tattooed individuals, that percent was 22.9% (p<0.001). Perceptions of the attractiveness of those with tattoos also differed by tattoo status (Figure 1): 10.2% of those without tattoos perceived those with tattoos as being more attractive, and 35.4% perceived them as less attractive. For those with tattoos, 26.0% saw those with tattoos as more attractive and 5.5% as less attractive than those without tattoos (p<0.001).

Additionally, those without tattoos were more likely to view individuals with tattoos as less intelligent (24.8%) and less professional (42.0%) than those with tattoos (4.9% and 15.0%, respectively; p<0.001).

Tattoo Characteristics

The average age when participants were first tattooed was 22.6 (SD 8.6) years (Table 4). The average time with tattoos was 15.4 (SD 11.2) years. The mean number of tattoos was 3.6 (SD 2.9). Tattoos were most common on the upper arms (41.5%), upper back/shoulder (35.4%), and lower arms (34.5%) (Table 4 and Appendix).

**Table 4:** Tattoo characteristics

Characteristics	With Tattoos (N=1072)
Age received first tattoo (years), mean (SD)	22.6 (8.6)
Time with tattoos (years), mean (SD)	15.4 (11.2)
Number of tattoos, mean (SD)	3.6 (2.9)
Location (N, %)	
Upper arms	445 (41.5)
Upper back/shoulder	380 (35.4)
Lower arms	370 (34.5)
Legs	245 (22.9)
Chest	215 (20.1)
Ankle	191 (17.8)
Lower back/buttocks	188 (17.5)
Face/neck	152 (14.2)
Hands/fingers/wrist	133 (12.4)
Feet/toes	114 (10.6)
Abdomen/stomach	100 (9.3)
Hips	94 (8.8)
Rib cage/side	32 (3.0)
Other	1 (0.1)
Reason for tattoo (N, %)	
Express myself	662 (61.8)
Fun	440 (41.0)
Remembrance	369 (34.4)
Enhance appearance	96 (9.0)
Peer pressure	34 (3.2)
Recommended	18 (1.7)
Wanted to/why not	16 (1.5)
Identity (tribal, family tradition, military)	15 (1.4)
Love/friendship	10 (0.9)
Other (medical alert, gift)	9 (0.8)
Mark a milestone (turned 50, grandchild)	4 (0.4)
Stupidity/drunk	3 (0.3)
To demonstrate ability to perform tattoos (yours or another person's)	2 (0.2)

When asked the reason for getting a tattoo, the most reported responses were to express themselves (61.8%), fun (41.0%), or remembrance (34.4%). Less common reasons for getting a tattoo included peer pressure (3.2%), love/friendship (0.9%), or to mark a milestone, such as turning 50 or the birth of a grandchild (0.4%).

Risk-taking Behaviors Among Those With Tattoos

Using the AUDIT-3 classification, 36.4% of those with tattoos were screened with either hazardous drinking or AUD (p<0.05, Table 5). The use of recreational drugs was reported by 16.1% of those with tattoos. Some individuals reported that they received a tattoo when intoxicated (11.5%) or high (8.8%). Approximately 25% of those with tattoos reported spending some time in jail (14.7% three or more days and 10.1% less than three days).

**Table 5:** Risk-taking behaviors of those with tattoos

Risk-taking behaviors	N=1072 (100.0%)
Intoxicated when tattooed, N (%)	
Yes	123 (11.5)
No	941 (87.8)
Not sure	8 (0.7)
High when tattooed, N (%)	
Yes	94 (8.8)
No	971 (90.6)
Not sure	7 (0.7)
Have you been in jail?, N (%)	
Yes, 0 to less than 3 days in jail	108 (10.1)
Yes, 3 or more days in jail	158 (14.7)
No	779 (72.7)
Prefer not to say	27 (2.5)
Alcohol consumption, N (%)	
No alcohol disorders	682 (63.6)
Hazardous drinking	135 (12.6)
Alcohol use disorder	255 (23.8)
Recreational drugs, N (%)	
Yes	173 (16.1)
No	864 (80.6)
Prefer not to say	35 (3.3)

Adverse Events Associated With Tattoos

Of those with tattoos, 451 (42.1%) reported one or more tattoo-related AEs. The most reported AE was pruritus (34.9%), followed by pain (14.7%), edema (13.1%), and infection (3.0%) (Table 6). Only 1.4% reported a primary care physician visit, and 1.0% reported an urgent care or emergency department visit for a tattoo-related AE.

**Table 6:** Adverse events around the tattoo site (N=1072)

Adverse event	N (%)
Infection	32 (3.0)
Pruritus/itching	374 (34.9)
Pain	158 (14.7)
Edema/swelling	140 (13.1)
Primary care physician visit due to tattoo adverse event	15 (1.4)
Emergency/urgent care visit due to tattoo adverse event	11 (1.0)

Tattoo Regret

Of those with tattoos, 18.2% reported regretting one or more of their tattoos. Those with regret reported having their tattoo for longer than those without regret (18.4 (SD 12.0) vs. 14.7 (SD 10.8) years; p<0.001). 

**Figure 2:**
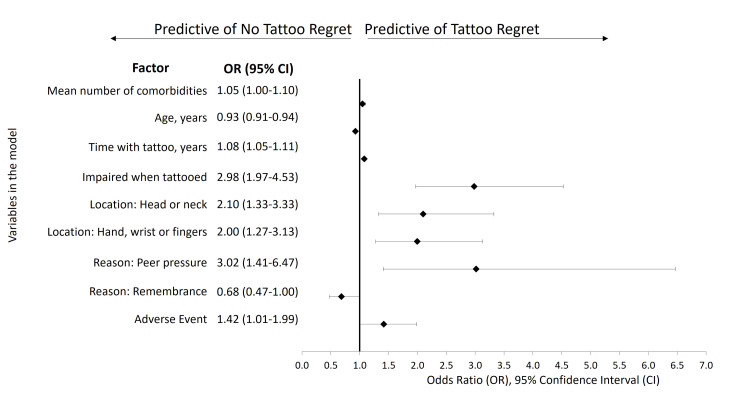
Factors predictive of tattoo regret

A logistic regression (Figure 2) found that the number of comorbid conditions (odds ratio (OR) 1.05), time with a tattoo (OR 1.08), being impaired when getting the tattoo (OR 2.98), having a tattoo on the head or neck (OR 2.10), having a tattoo on the hands, wrist or fingers (OR 2.00), peer pressure as the reason for getting the tattoo (OR 3.02), and experiencing a tattoo related AE (OR 1.42) were predictive of having tattoo regret. Older age (OR 0.93) and remembrance as the reason for the tattoo (OR 0.68) were predictive of not having tattoo regret.

As for future plans, 52.1% reported interest in removing, covering, or revising one or more of their tattoos, whereas 20.1% reported interest in another tattoo and 27.8% reported no interest in changing their tattoo status.

## Discussion

The prevalence of tattooed individuals in the US adult population has not been well documented in recent published literature. Harder yet to find are studies reporting reasons why individuals tattoo themselves or experience tattoo regret. There is general agreement in older studies that the trend for tattooing has been and is increasing, particularly among young adults. Studies on tattooing, as well as other forms of body art, also suggest that stigmas once associated with tattoos - crime, promiscuity, substance abuse - no longer hold true. Tattoos are more socially acceptable today than they have been in the past.

In 2019, the prevalence of US adults with tattoos was reported at 30% [2]. In 2023, Pew Research Center reported the prevalence of tattooed US adults at 32% [23]. Our study, which had a 2017 sample population with demographics including region, sex, race, and age, approximately similar to that of the US Census (Table 1), found that 35.3% of US adults had at least one tattoo. The consistency between the findings lends credibility to the perceptions of tattooing as a growing trend with gaining social acceptance and diminishing stigmas.

In our study, "self-expression" was far and away the most reported reason for getting tattooed (62%). "Fun" was the second most-reported reason at 20 points behind, and "remembrance" 7 points after that. Unsurprisingly, "remembrance" was also found to be predictive of not regretting a tattoo. Reasons that were perhaps more common for tattooing 20 or 30 years ago (e.g., military, tribal, tradition) were reported by less than 2% of our sample.

Our study found the most reported location of tattoos was the upper arm, closely followed by the upper back/shoulders and lower arms. These commonly reported tattoo locations are also locations that can easily be covered in professional settings. That said, given the changing views on tattoos, it is unclear whether tattoos need to be covered in all professional settings.

Drawing conclusions about tattoo perceptions is difficult because of the many variables that create a perception and the weight a person may give to each variable. Pew Research Center reported that non-tattooed individuals had more negative than positive impressions of tattooed individuals across sex, race, age, education, and income classifications, although the majority of respondents reported neither positive nor negative reactions [23]. However, Pew Research Center did not report impressions of tattooed individuals by tattooed individuals. As discussed in the introduction, negative perceptions about employment depend on the type of job, the customer base, the person interviewing, the type and location of the tattoo, the number of applicants, and so forth. Our study also found the majority of people - with and without tattoos - perceived tattooed and non-tattooed people equally in terms of rebelliousness, attractiveness, intelligence, and professionalism. However, our study showed a continued stigma among those without tattoos for their counterparts. Another way of framing these results is to consider that people tend to be more comfortable with, or think more highly of, others who are similar in appearance. Our research found tattooed individuals perceive other tattooed individuals - others similar in appearance to themselves - to be more attractive, more intelligent, and more professional than those dissimilar in appearance (i.e., non-tattooed). Conversely, the findings indicated that non-tattooed individuals perceived tattooed individuals as less attractive, less intelligent, and less professional.

Our study found that 18.2% of tattooed US adults regretted one or more of their tattoos, and that those with regret had had their tattoos longer. While the Pew Research Center reported regret at 24%, the age group with the highest proportion of regret were the oldest adults (65 years or more), and the representation of those 65 years or more with tattoos in our study was much lower at only 2.9% [23]. Also, our population was from 2017, and in the past six years, an increase in regret may be the companion to the trend of an increase in tattoos.

Not surprisingly, factors found to be predictive of tattoo regret were more time (in years) with a tattoo, impairment when tattooed, head or neck location, hands, wrist or fingers location, peer pressure as the reason, experiencing a tattoo-related AE, and higher number of comorbid conditions. Factors predictive of not having tattoo regret were also not surprising: older age and remembrance as the reason for the tattoo. The older the individual is, the more likely they are to know themselves and know what they want and can live with in terms of a tattoo. A tattoo that represents a loved and dearly missed person, place, or thing (e.g., animal) is likely to evoke a warm or positive memory and, therefore, less likely to be regretted.

To our knowledge, this is the first study to assess the prevalence and correlates of tattooing and tattoo regret among US adults. The large representative sample increases the probability of generalizability of the results. Limitations of this study include the inherent nature of self-reported survey research, participation limited to those with online access, and willingness to volunteer. Limitations of this study impact the generalizability of these results in samples with different characteristics.

## Conclusions

In this sample of US adults, more than one-third reported having a tattoo. While most people, regardless of their tattoo status, perceived tattooed and non-tattooed individuals equally, tattooed individuals were more likely to be perceived negatively than positively by those without tattoos. Whether tattooed or not, being aware of varying perceptions of tattoo status may be helpful in facilitating positive outcomes.

Individuals considering a tattoo or counseling someone considering a tattoo may find it helpful to know which factors can lead to regret. In general, an individual will be less likely to regret their tattoo if the tattoo location is not difficult to cover and their decision is not influenced by peers or by substance use (i.e., impaired judgment). They will be less likely to regret their decision the older they are when tattooed and if the reason for their tattoo is remembrance. It is important to point out that the longer an individual has a tattoo, the more likely they are to regret having the tattoo.

## Figures and Tables

**Figure 3:**
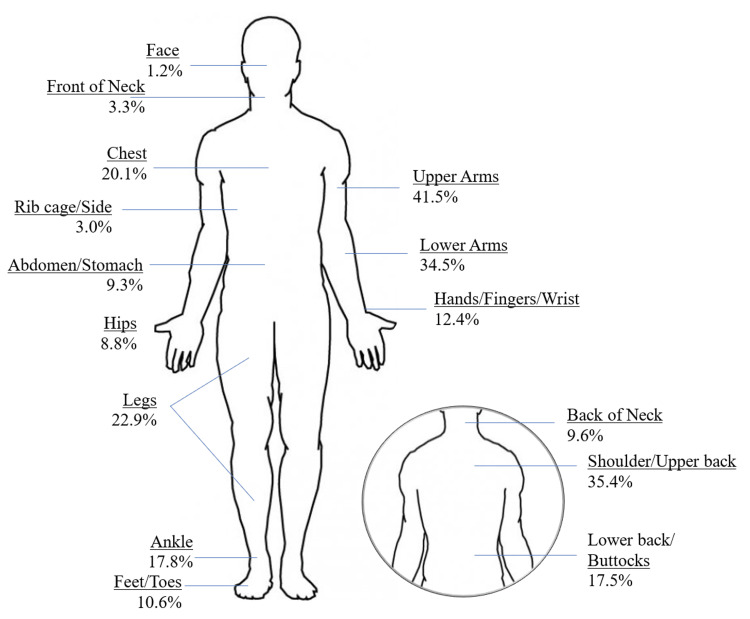
Location of tattoos Image source: "free download" http://getdrawings.com/medical-human-body-outline-drawing#medical-human-body-outline-drawing-27.jpg

## References

[1] Kluger N (2015). Epidemiology of tattoos in industrialized countries. Curr Probl Dermatol.

[2] (2019). More Americans have tattoos today than seven years ago. https://www.ipsos.com/sites/default/files/ct/news/documents/2019-08/tattoo-topline-2019-08-29-v2_0.pdf.

[3] Laumann AE, Derick AJ (2006). Tattoos and body piercings in the United States: a national data set. J Am Acad Dermatol.

[4] Swami V, Pietschnig J, Bertl B, Nader IW, Stieger S, Voracek M (2012). Personality differences between tattooed and non-tattooed individuals. Psychol Rep.

[5] Lande RG, Bahroo BA, Soumoff A (2013). United States military service members and their tattoos: a descriptive study. Mil Med.

[6] Madfis E, Arford T (2013). The dilemmas of embodied symbolic representation: regret in contemporary American tattoo narratives. J Soc Sci.

[7] Swami V (2011). Marked for life? A prospective study of tattoos on appearance anxiety and dissatisfaction, perceptions of uniqueness, and self-esteem. Body Image.

[8] Liszewski W, Kream E, Helland S, Cavigli A, Lavin BC, Murina A (2015). The demographics and rates of tattoo complications, regret, and unsafe tattooing practices: a cross-sectional study. Dermatol Surg.

[9] Rivera FP (2021). A highlight on reasons for tattoo regrets and removal. Medical Lasers.

[10] van Oosterzee AF (2009). Are you regretting your tattoo?. Enschede: University of Twente.

[11] Timming AR (2015). Visible tattoos in the service sector: a new challenge to recruitment and selection. Work, Employment and Society.

[12] French MT, Mortensen K, Timming AR (2019). Are tattoos associated with employment and wage discrimination? Analyzing the relationships between body art and labor market outcomes. Human Relations.

[13] Tews MJ, Stafford K, Kudler EP (2020). The influence of tattoo content on perceptions of employment suitability across the generational divide. J Person Psych.

[14] De Cuyper C (2020). How to advise a patient who wants a tattoo?. Presse Med.

[15] Rahimi IA, Eberhard I, Kasten E (2018). Tattoos: what do people really know about the medical risks of body ink?. J Clin Aesthet Dermatol.

[16] Otto MA (2015). Teenage tattoos are most often regretted. http://teenage-tattoos-are-most-often-regretted.

[17] Dukes RL (2016). Regret among tattooed adolescents. J Soc Sci.

[18] Selim AJ, Rogers W, Fleishman JA, Qian SX, Fincke BG, Rothendler JA, Kazis LE (2009). Updated U.S. population standard for the Veterans RAND 12-item Health Survey (VR-12). Qual Life Res.

[19] Kroenke K, Spitzer RL, Williams JB (2001). The PHQ-9: validity of a brief depression severity measure. J Gen Intern Med.

[20] Spitzer RL, Kroenke K, Williams JB, Löwe B (2006). A brief measure for assessing generalized anxiety disorder: the GAD-7. Arch Intern Med.

[21] Reinert DF, Allen JP (2007). The alcohol use disorders identification test: an update of research findings. Alcohol Clin Exp Res.

[22] Kazis LE, Selim AJ, Rogers W, Qian SX, Brazier J (2012). Monitoring outcomes for the Medicare Advantage program: methods and application of the VR-12 for evaluation of plans. J Ambul Care Manage.

[23] Schaeffer K, Dinesh S (2023). 32% of Americans have a tattoo, including 22% who have more than one. Pew Research Center.

